# Rapid Amygdala Gamma Oscillations in Response to Eye Gaze

**DOI:** 10.1371/journal.pone.0028188

**Published:** 2011-11-30

**Authors:** Wataru Sato, Takanori Kochiyama, Shota Uono, Kazumi Matsuda, Keiko Usui, Yushi Inoue, Motomi Toichi

**Affiliations:** 1 The Hakubi Project, Kyoto University, Inuyama, Aichi, Japan; 2 Faculty of Human Health Science, Kyoto University, Kyoto, Kyoto, Japan; 3 National Epilepsy Center, Shizuoka Institute of Epilepsy and Neurological Disorders, Shizuoka, Shizuoka, Japan; Cuban Neuroscience Center, Cuba

## Abstract

**Background:**

The eye gaze of other individuals conveys important social information and can trigger multiple psychological activities; some of which, such as emotional reactions and attention orienting, occur very rapidly. Although some neuroscientific evidence has suggested that the amygdala may be involved in such rapid gaze processing, no evidence has been reported concerning the speed at which the amygdala responds to eye gaze.

**Methodology/Principal Findings:**

To investigate this issue, we recorded electrical activity within the amygdala of six subjects using intracranial electrodes. Subjects observed images of eyes and mosaics pointing in averted and straight directions. The amygdala showed higher gamma-band oscillations for eye gaze than for mosaics, which peaked at 200 ms regardless of the direction of the gaze.

**Conclusion:**

These results indicate that the human amygdala rapidly processes eye gaze.

## Introduction

The eye gaze of other individuals triggers multiple psychological activities in the observer. Some, such as attention orienting [1], [2] and emotional reactions [3], occur rapidly and automatically, whereas others, such as reading others' mental states [4], occur slowly and intentionally. These processes have been shown to play important roles in real-life interactions (cf. [5]) and in clinical disorders (cf. [6]).

However, the neural substrates of gaze processing, particularly of the rapid sort, remain unknown. Some neuroimaging [7]–[10] and neuropsychological [11]–[13] studies have shown involvement of the amygdala in the processing of eye gaze. Because the amygdala receives input from subcortical as well as neocortical visual pathways [14], some researchers have speculated that amygdala activation may occur at an early stage of gaze processing (e.g., [12]). However, the amygdala has also been shown to be involved in the slow processing of visual stimuli, which takes several seconds or more (cf. [15]). No definite information about how fast the human amygdala responds to eye gaze has been reported.

**Figure 1:**
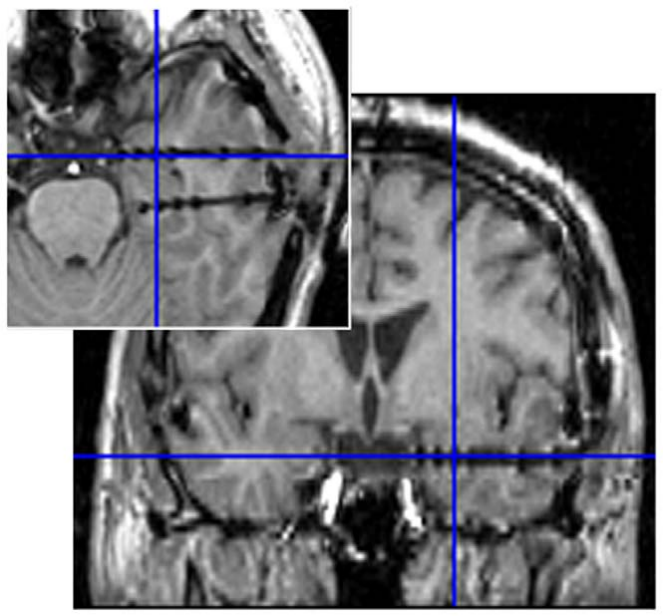
Representative anatomical magnetic resonance images. Blue crosses indicate the location of the amygdala electrodes.

**Figure 2:**
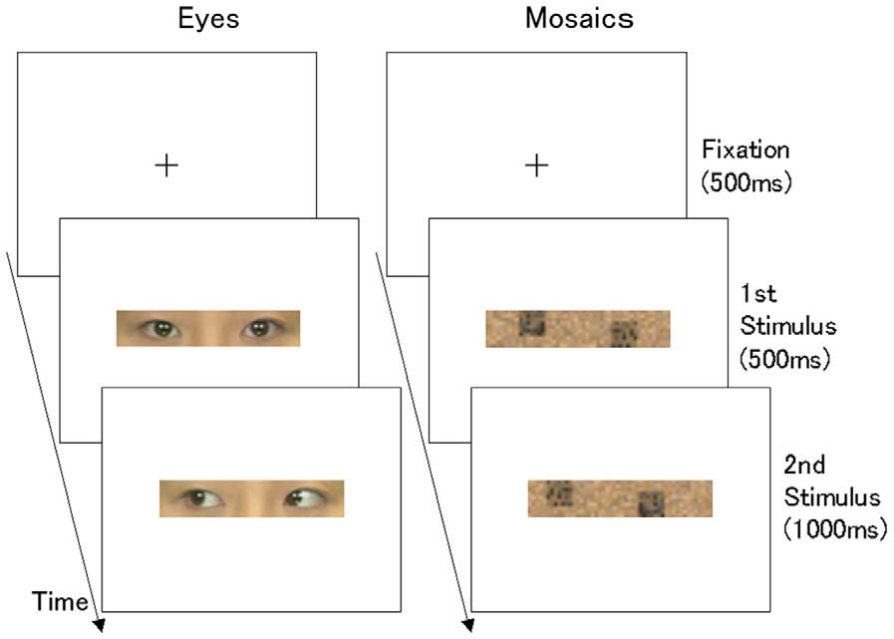
Illustrations of stimulus presentations. Straight–averted direction conditions of eyes and mosaic stimuli are shown.

To test the speed of the human amygdala response to eye gaze, we recorded the electric field potential activities of the human amygdala using intracranial electrodes in six subjects undergoing pre-neurosurgical assessment (Fig. 1). The subjects were presented with visual stimuli showing only the eye region (Fig. 2). To examine the effect of the direction of the eye gaze, both averted and straight gazes were presented. Control stimuli were mosaic patterns, constructed from fragments of the original gaze stimuli and thus characterized by the same brightness, indicative of the straight or averted direction. To test amygdala activity in response to dynamic changes in gaze direction, the second set of stimuli was the reverse of the first (i.e., averted if the first gaze was straight or straight if it was averted); these stimuli were presented 500 ms after the onset of the first stimuli. Amygdala field potential data were analyzed using time–frequency statistical parametric mapping (SPM) [16]. To confirm the spatial specificity of the recorded field potentials related to anatomical locations, we analyzed the electrodes located in the amygdala as well as in the adjacent white matter. Due to debate about the contamination effect of the electrical activities of ocular muscles on intracranial field potentials (e.g., [17], [18]), we also recorded and analyzed electrooculogram (EOG).

## Methods

### Ethics Statement

This study was approved by the local ethics committee of the Shizuoka Institute of Epilepsy and Neurological Disorders.

### Subjects

Six patients (five females and one male; mean ± *SD* age, 34.5±7.9 years) participated in the experiment. All were suffering from pharmacologically intractable focal epilepsy, and intracranial electrodes were implanted as part of a presurgical evaluation. The experiment was conducted 2.0–2.8 weeks after electrode implantation while the subjects were participating in a series of neuropsychological and electrophysiological assessments (e.g., [19]).

Neuropsychological assessments confirmed that all subjects' language ability and everyday memory were intact. During the experiment, no seizure was observed, and all subjects were mentally stable. All subjects were right-handed, as assessed using the Edinburgh Handedness Inventory [20]. All had normal or corrected-to-normal visual acuity. All subjects gave written informed consent after the procedure was fully explained.

### Anatomical MRI assessment

Pre- and post-implantation anatomical assessments were conducted using the structural magnetic resonance imaging (MRI) on a 1.5-T scanning system (Signa Twin Speed, General Electric Yokokawa) using T-1 weighted images. Pre-implantation MRI assessments and surgical evaluations showed no structural abnormalities in the bilateral amygdala of any subject.

Implantation of intracranial electrodes was performed using the stereotactic method [21]. Implantation sites were chosen solely based on clinical criteria. To record the activities of the bilateral amygdalae, six electrodes were implanted horizontally in each hemisphere. Post-implantation anatomical MRI assessments confirmed that the third (numbered from the medial to the lateral side) electrodes were implanted in the bilateral amygdala in all subjects (Fig. 1). The assessments also showed that the fifth electrodes were located in the white matter adjacent to the anterior temporal cortex. A probabilistic cytoarchitectonic map of the amygdala [22] was also referenced to validate our selections.

### Stimuli

Stimuli (Fig. 2) were prepared using MATLAB 6.5 (Mathworks). Eyes stimuli were created from color photographs of full-face neutral expressions displayed by four females and three males, looking either to the left or straight ahead. Only the eyes were cut from the photographs; no other facial features and no eyebrows were visible in the stimuli. Mirror images of these stimuli were created. Eyes looking left or right were used for the averted direction condition, and eyes looking straight ahead were used for the straight direction condition.

The mosaic stimuli were constructed from the eyes stimuli. First, all of the eyes stimuli were divided into small squares (10 vertical×50 horizontal), and all squares were set to the mean brightness of pixels in each square. To construct objects indicating directional information in the manner of the eyes stimuli, two sets of 49 small squares with the highest brightness were selected and randomly arranged to construct two large diagonally aligned squares. The squares were aligned diagonally because our preliminary experiment indicated that large squares arranged horizontally looked like eyes. The horizontal center of these large squares was comparable to the pupil positions of the eyes stimuli. Other small squares were then randomly arranged in other areas. These manipulations resulted in mosaic stimuli equivalent to the corresponding original eyes stimuli in terms of overall brightness and directional information, with no eye features incorporated.

The mean brightness of the images was made constant. Stimuli with different direction conditions were shown for the first and second stimulus presentations to represent directional changes.

### Procedure

The events were controlled by SuperLab Pro 2.0 (Cedrus) and implemented on a Windows computer (FSA600, Teknos). Stimuli were presented on a 19-inch cathode ray tube monitor (GDM-F400, Sony) with a refresh rate of 100 Hz and a resolution of 1024×768 pixels. Subjects' responses were recorded using a response box (RB-400, Cedrus).

Subjects were tested individually in a quiet room. Subjects were comfortably seated with their heads supported by a chin-and-forehead rest 0.57 m from the monitor. The resulting visual angle subtended by the stimulus was 1.5° vertically×7.5° horizontally.

Each stimulus was presented three times. In addition, a red cross was presented as the target in 15 trials. Thus, each subject performed 183 trials (42 trials each of averted eyes–straight eyes, straight eyes–averted eyes, averted mosaics–straight mosaics, and straight mosaics–averted mosaics, as well as the 15 target trials). The stimuli were presented in random order. In each trial, after the presentation of a cross-shaped fixation point for 500 ms, the first stimulus was presented for 500 ms in the center of the visual field. Then the second stimulus was presented for 1000 ms. In each target trial, instead of eyes or mosaic stimuli, the red cross was presented until a response was made. The subjects were instructed to press a button with their right forefingers as quickly as possible after detecting the red cross. This task ensured that subjects kept their attention on the stimuli and it prevented the explicit processing of eye gaze. Post-hoc debriefing confirmed that the subjects were not aware that the purpose of the experiment was the investigation of gaze processing. The subjects were instructed not to blink while the stimuli were being presented. Intertrial intervals were randomly varied between 2000 and 5000 ms. To avoid habituation and drowsiness, subjects were given short breaks every 45 trials. Before data collection began, subjects were familiarized with the procedure using a block of 10 training trials.

### Data recording

Intracranial field potential recording was conducted using depth platinum electrodes (0.8 mm in diameter; Unique Medical). All electrodes were referenced to the electrodes (2.3 mm in diameter; Ad-tech) embedded within the scalp of the midline dorsal frontal region. Impedances were balanced and maintained below 5 kΩ. Data were amplified, filtered online (band pass: 0.5–120 Hz; notch: 60 Hz), and sampled at 1000 Hz by an electroencephalograph system (EEG-1100, Nihon-Koden). Vertical and horizontal EOGs were simultaneously recorded using Ag/AgCl electrodes (Nihon-koden). A video recording was unobtrusively conducted using a video camera attached to the electroencephalograph. Offline checks of the videos confirmed that all subjects were engaged throughout all tasks.

### Data analysis

Intracranial recording data were re-sampled using Psychophysiological Analysis Software 3.3 (Computational Neuroscience Laboratory of the Salk Institute) implemented in MATLAB 6.5 (Mathworks). The data were sampled for 1500 ms in each trial, which consisted of pre-stimulus baseline data for 500 ms (the fixation point was presented) and the data for 1000 ms after stimulus onset at sampling rate of 200 Hz. Any epoch whose amplitude was beyond the total mean±3 SD for each electrode in each subject was rejected as an artifact. The frequencies of artifact-contaminated epochs for the amygdala electrodes were 11.9 and 11.0% for the first and second stimulus presentations, respectively. No systematic differences among the conditions related to the occurrence of artifacts (four-way analysis of variance, *p* >0.1) were found.

Time–frequency analyses were performed using SPM5 (http://www.fil.ion.ucl.ac.uk/spm/) implemented in MATLAB 6.5 (Mathworks). First, time–frequency (power) maps were calculated for each trial using a continuous wavelet decomposition with seven-cycle Morlet wavelets from 4 to 60 Hz, which covered theta (4–8 Hz), alpha (8–12 Hz), beta (12–30 Hz), and gamma (30–60 Hz) activity. To enhance Gaussianity, the time–frequency maps were then log-transformed and smoothed with a 2D Gaussian kernel of full width at half-maximum of 12 Hz in the frequency domain and 96 ms in the time domain [16].

The time–frequency maps were entered into the general linear model (GLM) based on a fixed-effects analysis of the pooled error from all trials of all subjects. Separate analyses were conducted for the first and second stimulus presentations. The GLM included stimulus type (eyes, mosaics), direction (averted, straight), and laterality (left, right) as factors of interest, and subject blocks (six subjects) as a factor of no interest. We analyzed the main effects of stimulus type (eyes versus mosaics) and interactions related to the stimulus type factor, using one-dimensional linear contrast. The time–frequency SPM{*T*} was calculated for each contrast. To ensure the assumption of independent and identically distributed error in the GLM, a correction for non-sphericity was applied using the restricted maximum likelihood procedure [23]. To validate the cluster-size inference, we also performed a correction for non-stationary smoothness in the time–frequency random field using the VBM5.1 Toolbox (http://dbm.neuro.uni-jena.de/vbm/) [24].

Statistical inference on the time–frequency SPM{*T*} was based on random field theory [25]. The analyses were conducted during the 500 ms following the first or second stimulus presentation and within a frequency range of 4–60 Hz. Significantly activated clusters were identified if they reached the extent threshold of *p* <0.05 (corrected for multiple comparisons), with the height threshold of *p* <0.01 (uncorrected). To confirm the consistency of the effects across subjects, we conducted conjunction analyses based on a global null hypothesis [26] with the height threshold of *p* <0.01 (uncorrected).

For display purposes, adjusted time–frequency maps were calculated. The grand mean activity across all conditions and the subject effects were covaried out as an effect of no interest. Additionally, the effect-size data were extracted from the time–frequency maps at activation foci. The data were sampled using the rectangular window, which extended to 30 ms in the time dimension and 6 Hz in the frequency dimension.

The data obtained by the electrodes in the adjacent white matter were also analyzed using the same procedures used in the time–frequency SPM analyses for the amygdala.

We conducted two types of follow-up analyses on the EOG data to test the possible contaminating effect of the electrical activities of ocular muscles on amygdala activity. First, we analyzed correlations between the trains of epoched data on amygdala activity and on horizontal or vertical EOGs. Because the gamma-band activity was found to be relevant in the above analyses, we also analyzed these correlations after gamma-band filtering. Correlation coefficients were evaluated for differences from zero by one-sample *t*-tests after Fisher's *r*-to-*z* transformation. Next, we conducted the above time–frequency SPM analyses for amygdala activity with the nuisance covariates of the gamma-band amplitudes of horizontal and vertical EOGs.

## Results

The performance on dummy target detection was perfect (correct identification rate  = 100.0%) and showed no delays in reaction times (mean ± *SD* = 261.0±15.6 ms; range 183–389 ms).

**Figure 3:**
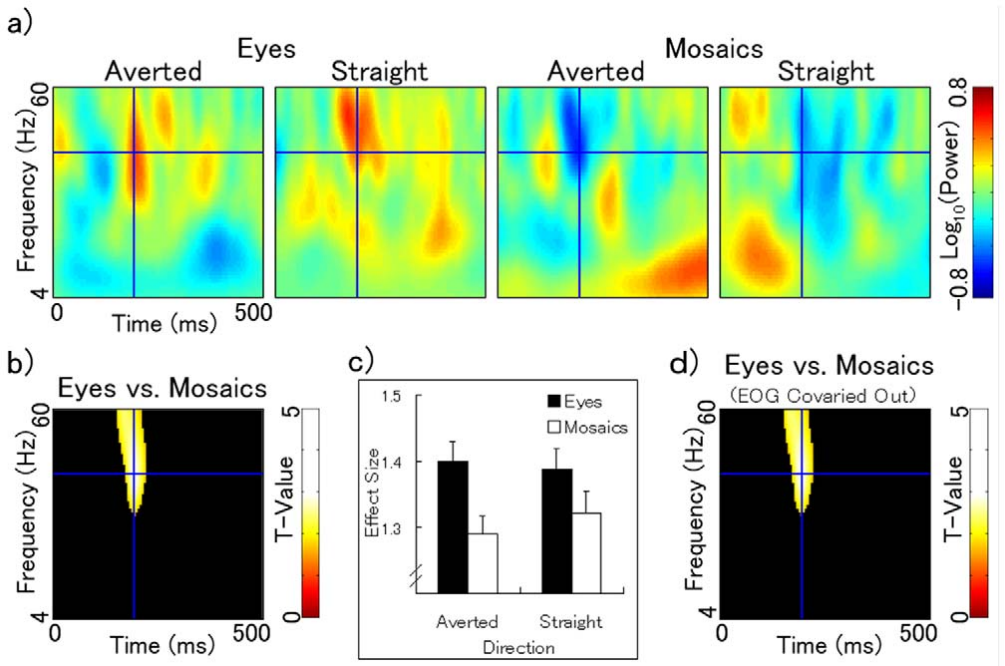
Amygdala activity under the first stimulus presentation condition. a) Adjusted time–frequency maps of the amygdala for averted eyes, straight eyes, averted mosaics, and straight mosaics under the first stimulus presentation condition. The results for both hemispheres are combined. Blue crosses indicate the locations of activation foci for the main effects of stimulus type, contrasting the effects of eyes versus mosaics (200 ms, 44 Hz). b) Statistical parametric maps that exhibited higher activation for eyes than for mosaics. A blue cross indicates the location of activation focus. c) Mean (with SE) effect size at the peak activation focus of the amygdala. The results of both hemispheres are combined. d) Statistical parametric maps that exhibited higher activation for eyes than for mosaics in the analyses after covarying out the gamma-band horizontal and vertical EOGs. A blue cross indicates the location of activation focus.

The intracranial field potential data for the amygdala were subjected to wavelet decomposition, and the resultant time–frequency maps were analyzed with models that included the effects of stimulus type, direction, and laterality. Separate analyses were conducted for the first and second presentations. The contrast of eyes versus mosaics for the first stimulus presentations revealed significant gamma-band activity peaking at 200 ms and 44 Hz (*Z* = 4.41; Fig. 3a–c). The same contrast for the second stimulus presentations also showed some activation peaking at 285 ms and 40 Hz (*Z* = 2.72), although it failed to reach significance with respect to the extent threshold (Fig. S1a–c). Conjunction analyses confirmed activations in these clusters. The main effect of stimulus type did not show any other evident activation in whole time–frequency regions (4–60 Hz, 0–500 ms) for either the first or the second stimulus presentation. Two- and three-way interactions related to stimulus type also revealed no significant effect on activation.

To test the spatial specificity of the activities observed in the amygdala, the data obtained from the electrodes in the adjacent white matter were also analyzed. Visual inspections of the time–frequency maps (Fig. S2) revealed that the activation patterns of these electrodes differed from those in the amygdala. Statistical analyses revealed no significant main effects or interactions for either the first or second presentation.

To test the possible contaminating effect of the electrical activities of ocular muscles on amygdala activity, we first analyzed the correlations between amygdala activity and horizontal or vertical EOGs. The results showed no significant correlation (mean ± *SD r* = 0.05±0.12, −0.12±0.29, 0.06±0.15, and −0.09±0.33 for the left amygdala–horizontal EOG, left amygdala–vertical EOG, right amygdala–horizontal EOG, and right amygdala–vertical EOG, respectively; *p*s >0.1). Non-significant correlations were also found when the gamma-band activities were analyzed (mean ± *SD r* = 0.10±0.25, −0.01±0.34, 0.11±0.26, and 0.02±0.37 for the left amygdala–horizontal EOG, left amygdala–vertical EOG, right amygdala–horizontal EOG, and right amygdala–vertical EOG, respectively; *p*s >.10). The analyses of amygdala activity were conducted again after covarying out the gamma-band horizontal and vertical EOGs. Identical main effects of stimulus type were confirmed for the first and second stimulus presentations (Fig. 3d; Fig. S1d).

## Discussion

The gamma-band activity of the amygdala, which peaked at 200 ms, was more pronounced in response to eyes than to mosaics. The finding of the involvement of the amygdala in gaze processing is consistent with results of previous neuroimaging studies. Some neuroimaging studies have revealed that the amygdala is active while individuals view both straight [7] and averted [8] eye gazes. A neuropsychological study also showed that brain damage involving the amygdala resulted in impaired recognition of eye-gaze direction, leading to a failure to discriminate between faces looking toward and those looking away from the subject [13]. Furthermore, some evidence suggests rapid activation of the amygdala in response to gaze. For example, a neuroimaging study reported activation of the amygdala in response to eye-gaze stimuli without conscious awareness [9]. Other neuropsychological studies have shown that amygdala-damaged patients were impaired in terms of their reflexive attention orienting in response to eye gaze [11], [12]. However, due to methodological limitations in these studies, specific temporal information relating to gaze processing in the amygdala has, thus far, remained unknown. To the best of our knowledge, the present study is the first to demonstrate that the amygdala is involved in rapid eye gaze processing, specifically at 200 ms after stimulus onset.

Our results also showed a trend suggesting that the amygdala was active in response to a second stimulus presented at 200–300 ms. We assumed that the sequential presentations of stimuli showing different gaze directions induced apparent motion (cf. [27]), implementing dynamic changes in gaze direction. This interpretation is in line with the results of a previous neuroimaging study reporting that the amygdala was activated in response to video clips of gaze direction shifts [10]. Based on these data, we speculate that the amygdala may rapidly process not only the presence of gaze but also changes in gaze direction.

Because the participants were engaged in dummy tasks, the observed rapid amygdala activity could be regarded as primarily reflecting automatic gaze processing. The finding of rapid and automatic activity in the amygdala in response to eye gaze is consistent with behavioral evidence. Several previous studies have revealed that another individual's averted eye gaze reflexively triggers attention orienting (e.g., [1]), even without conscious awareness [28]. The straight gaze has also been shown to automatically draw attention [2]. Another line of research has shown that eye contact automatically induces subjective and physiological emotional reactions [3]. We speculate that the gamma oscillations in the amygdala at 200 ms may constitute one of the neural underpinnings of such automatic rapid psychological activities in response to another person's eye gaze.

Gaze-specific activity was not evident in the electrodes adjacent to the amygdala. This result is consistent with previous technical reports indicating that intracranial field potential recordings may have a spatial resolution of about a 1-cm radius [17], [29]. These data suggest that gaze-specific activity in the amygdala does not reflect the spillover activation of adjacent brain regions.

We found no significant correlations between amygdala activity and horizontal or vertical EOGs, and significant amygdala activity was observed after statistically controlling for the EOGs. Although debate about the contamination effect of the electrical activities of ocular muscles on intracranial field potentials persists (e.g., [17], [18]), these data, as well as the different patterns produced by the electrodes in the adjacent white matter, suggest that the present amygdala activity was free from systematic contamination by eye-movement artifacts.

In this study, we contrasted eyes versus mosaic stimuli, and such control of brightness reduced the possibility that amygdala activity reflected basic sensory processes. This notion is consistent with previous animal neurophysiological and human neuroimaging studies (e.g., [30]). However, the specific factors in the eyes that elicited the differences in amygdala activity remain to be specified. As mentioned above, eyes can induce various types of psychological activities. Ample evidence from neuropsychological and neuroimaging studies has also indicated that the amygdala is involved in multiple social and emotional functions (cf. [31]). Further studies are necessary to specify the nature of the information processing that occurs in the amygdala in response to eye gaze.

## Supporting Information

Figure S1 Amygdala activity under the second stimulus presentation condition. a) Adjusted time–frequency maps of the amygdala for averted eyes, straight eyes, averted mosaics, and straight mosaics. The results for both hemispheres are combined. Blue crosses indicate the locations of activation foci for the main effects of stimulus type, contrasting the effects of eyes versus mosaics (285 ms, 40 Hz). b) A statistical parametric map that exhibited evident activation for the main effects of stimulus type. A blue cross indicates the location of activation focus. c) Mean (with SE) effect size at the peak activation focus for the main effects of stimulus type. The results of both hemispheres are combined. d) Statistical parametric maps that exhibited higher activation for eyes than for mosaics in the analyses after covarying out the gamma-band horizontal and vertical EOGs. A blue cross indicates the location of activation focus.
(TIF)

Figure S2 Adjusted time–frequency maps of the white matter adjacent to the amygdala in response to averted eyes, straight eyes, averted mosaics, and straight mosaics under the first (a) and second (b) stimulus-presentation conditions. The results for both hemispheres are combined.
(TIF)
