# Supplementary figures and images for: Rapid Amygdala Gamma Oscillations in Response to Eye Gaze
Source: PLoS One. 2011 Nov 30;6(11):e28188. doi: 10.1371/journal.pone.0028188 (PMC3227649; doi:10.1371/journal.pone.0028188)

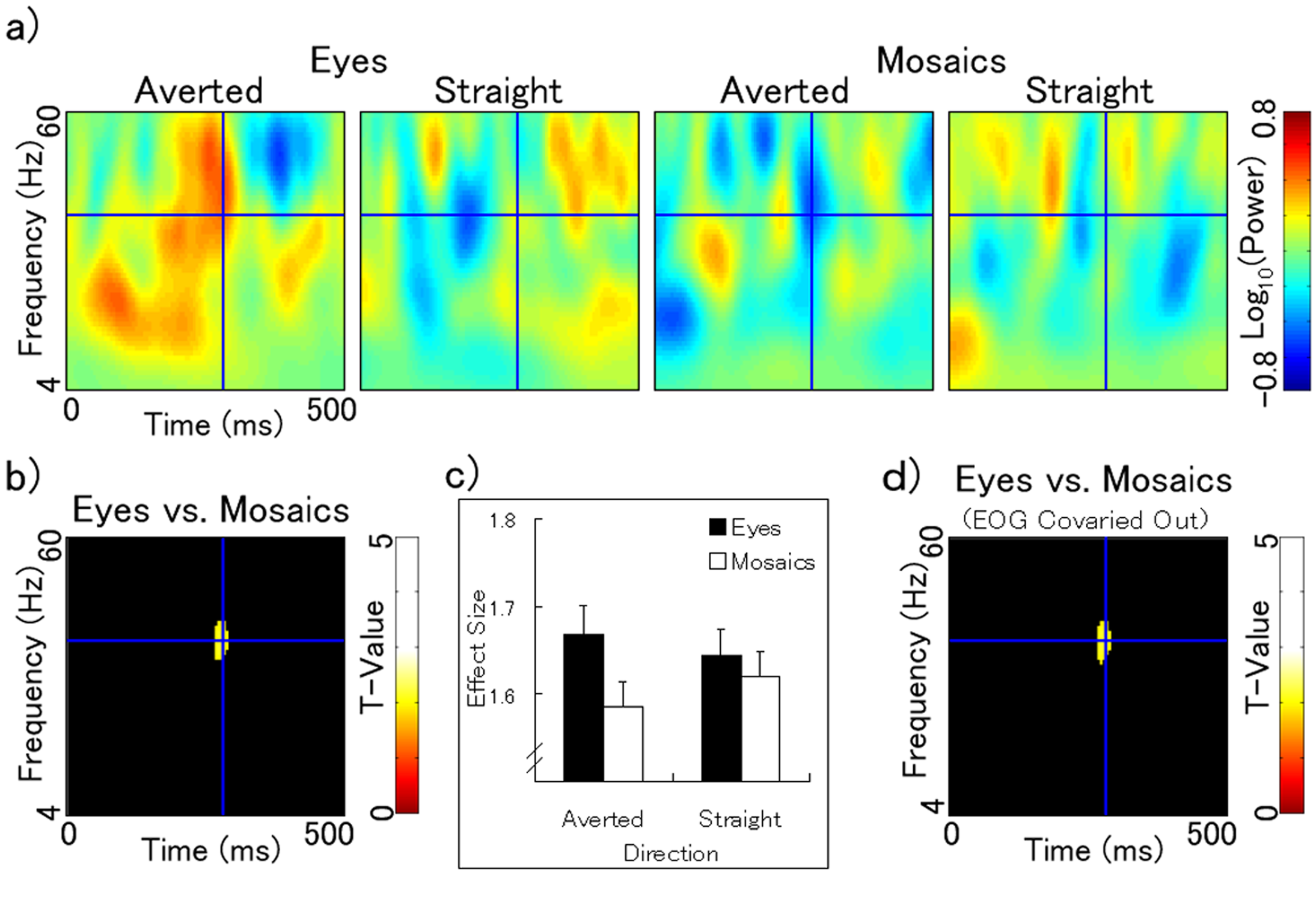

Supplement: Figure S1 — Amygdala activity under the second stimulus presentation condition. a) Adjusted time–frequency maps of the amygdala for averted eyes, straight eyes, averted mosaics, and straight mosaics. The results for both hemispheres are combined. Blue crosses indicate the locations of activation foci for the main effects of stimulus type, contrasting the effects of eyes versus mosaics (285 ms, 40 Hz). b) A statistical parametric map that exhibited evident activation for the main effects of stimulus type. A blue cross indicates the location of activation focus. c) Mean (with SE) effect size at the peak activation focus for the main effects of stimulus type. The results of both hemispheres are combined. d) Statistical parametric maps that exhibited higher activation for eyes than for mosaics in the analyses after covarying out the gamma-band horizontal and vertical EOGs. A blue cross indicates the location of activation focus. (TIF) [file pone.0028188.s001.tif]

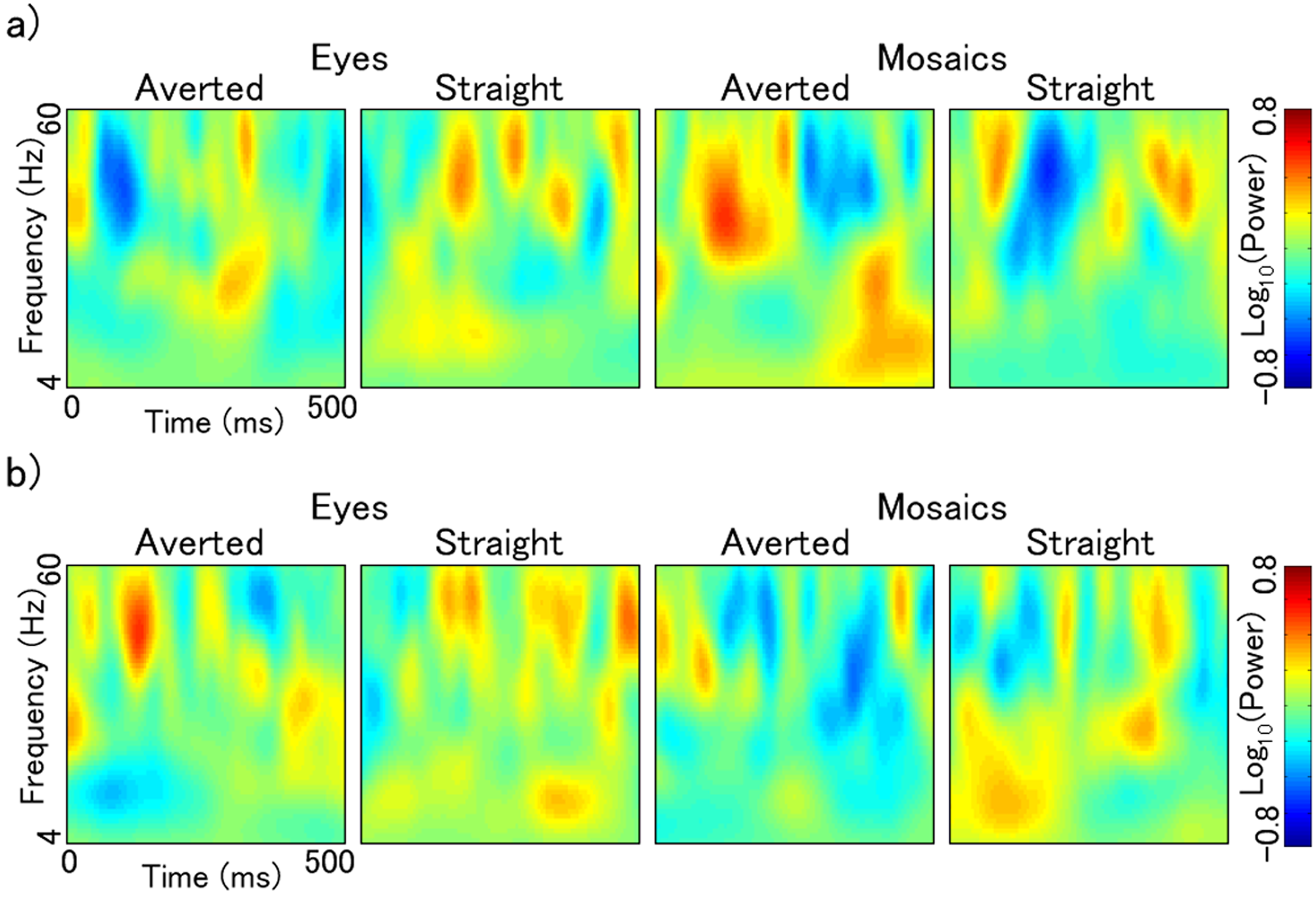

Supplement: Figure S2 — Adjusted time–frequency maps of the white matter adjacent to the amygdala in response to averted eyes, straight eyes, averted mosaics, and straight mosaics under the first (a) and second (b) stimulus-presentation conditions. The results for both hemispheres are combined. (TIF) [file pone.0028188.s002.tif]
